# Multimodal Chronic Pain Therapy for Adults via Smartphone: Randomized Controlled Clinical Trial

**DOI:** 10.2196/36114

**Published:** 2022-05-11

**Authors:** Yolanda Morcillo-Muñoz, Antonio José Sánchez-Guarnido, Silvia Calzón-Fernández, Isabel Baena-Parejo

**Affiliations:** 1 Primary Care Andalusian Health Service Algeciras Spain; 2 Primary Care Andalusian Health Service Motril Spain; 3 Primary Care Andalusian Health Service Sevilla Spain; 4 Primary Care Andalusian Health Service Córdoba Spain

**Keywords:** chronic pain, eHealth, multimodal intervention, catastrophizing, self-management, mHealth, mobile phone, randomized controlled trials

## Abstract

**Background:**

Combination therapies delivered remotely via the internet or mobile devices are increasingly being used to improve and promote the self-management of chronic conditions. However, little is known regarding the long-term effects of these interventions.

**Objective:**

The aim of this study is to evaluate the effectiveness of a multimodal intervention program that measures associated variables such as catastrophizing, pain acceptance, and quality of life using a mobile device in people with chronic pain in an outpatient setting.

**Methods:**

A randomized controlled clinical trial was performed using parallel treatment groups. A total of 209 patients with chronic musculoskeletal pain were randomly assigned to one of the two study arms. The intervention group received a standard *web-based* psychosocial therapy-type program of activities through a smartphone for 6 weeks. The control group only had access to the *Find out more* section of the app, which contained audiovisual material for pain management based on a self-help approach. The primary outcome was catastrophizing measured using the Pain Catastrophizing Scale (PCS). Secondary outcomes were pain acceptance measured using the Chronic Pain Acceptance Questionnaire and health-related quality of life measured using the EuroQol Visual Analogue Scale. Assessments were conducted at baseline (T1), after treatment (T2), and at the 3-month follow-up (T3). The variations between the different phases were assessed using the percentage change rescaled with log base 2. The Cohen *d* was calculated based on the results of the linear mixed model. The investigators of the study who evaluated the results were not involved in patient recruitment and were blinded to the group assignment.

**Results:**

Positive effects were found in the intervention group (T2–T1) in catastrophizing between the baseline and posttreatment phases (*P*<.001) and in helplessness (−0.72 vs 0.1; *P*=.002), rumination (−1.59 vs −0.53; *P*<.001), acceptance (0.38 vs 0.05; *P*=.001), and quality of life (0.43 vs −0.01; *P*=.002), although no significant changes were found for magnification (0.2 vs 0.77; *P*=.14) and satisfaction with health (0.25 vs −0.27; *P*=.13). Three months after treatment, significant differences were observed in the intervention group for the outcome variable of catastrophizing (PCS; −0.59 vs 0.2; *P*=.006) and the PCS subscales of helplessness (−0.65 vs 0.01; *P*=.07), rumination (1.23 vs −0.59; *P*=.04), and magnification (0.1 vs 0.86; *P*=.02).

**Conclusions:**

The results of our study suggest that app-based mobile multidimensional treatments for adults with chronic pain improve catastrophizing, quality of life, and psychological flexibility immediately after treatment and that the effects are maintained for the primary outcome of catastrophizing for at least 3 months following treatment. Moreover, they promote self-management and can be used to complement face-to-face pain treatments.

**Trial Registration:**

ClinicalTrials.gov NCT04509154; https://clinicaltrials.gov/ct2/show/NCT04509154

## Introduction

### Background

Pain is estimated to be among the top 10 conditions with the highest burden on health expenditure and health care resources and has a significant impact on patients’ quality of life [1,2]. Pain is defined as an “unpleasant sensory and emotional experience associated with or similar to that associated with actual or potential tissue damage” [3]. When it affects one or more anatomical regions; persists for >3 months; and is associated with emotional (anxiety or depressed mood) and functional distress that interferes with work, social, and family life, pain is considered chronic [4]. Although not a frequent cause of mortality in itself, many people die experiencing pain, and even more people are living with pain [5,6]. Owing to the extremely high prevalence of chronic pain in the general population, it should be considered a health problem.

There is a large body of research on multidisciplinary treatment programs for adults with chronic pain, including reviews of the clinical evidence, effectiveness of pain treatments, and cost-effectiveness of chronic pain programs in outpatient settings [7,8]. A systematic review by Hauser et al [9] reported that multidisciplinary programs are effective in reducing chronic pain and improving patients’ biopsychosocial situations and may also reduce the use of prescription medications. Similarly, the Canadian Agency for Drugs and Technologies in Health found that both multimodal therapy and monotherapy were beneficial for treating chronic pain. However, further research is needed to determine which type or combination of therapies can provide long-term benefits for these patients [8,10,11].

As chronic pain is a complex and multidimensional problem, it cannot be managed using medical therapies alone. Therefore, multidimensional treatments involving psychological therapies such as acceptance and commitment therapy (ACT), mindfulness, physical exercise, and health assets could play an important role in mitigating catastrophizing, improving pain acceptance, and reducing the use of psychotropic medications [8,12-14].

The available evidence supports the efficacy of several interventions for the self-management of chronic pain in outpatients. Some of the key components of these interventions are the administration of chronic pain medications according to the type of pain and patient comorbidities [7,10,14,15]; therapeutic exercise and patient education for the treatment of a wide range of musculoskeletal disorders [5,16,17]; patient education and counseling [18,19]; ACT as an evidence-based treatment for chronic pain intensity and depression [20,21]; mindfulness [22] to moderate the impact of catastrophizing on everyday pain [23,24]; and self-management [15,25,26]. Balancing activities with rest, stress management, emotion regulation, and appropriate physical exercise can also improve the quality of life of these patients [27]. An essential part of treatment is the early detection of catastrophic thinking, psychological inflexibility, and depression as the modification of these factors can reduce disability, decrease pain interference and intensity, and improve the ability of patients with chronic pain to perform activities of daily living [28].

In addition to face-to-face interventions for chronic conditions, interventions are increasingly being delivered via mobile apps or the internet as these technologies have become an essential part of people’s daily lives and are always on call. In a systematic review of the use of information and communication technologies (ICTs) in chronically ill patients, 15.4% of the studies found that ICTs had a positive impact on patient empowerment or self-management, 14% showed an improvement in physical conditions and quality of life, and 5.1% reported greater self-efficacy for managing disease [29-32]. In the field of health, new technologies are being used for a variety of purposes, among them symptom assessment, psychoeducation, and treatment adherence. Such technologies have been shown to be beneficial for the provision of health care as they can improve patient accessibility and health care response, are instantaneous, and occur in real time [33-35].

### Objectives

The aim of this study was to evaluate the effectiveness of a multimodal intervention program using a web-based smartphone or mobile device app. The app assesses pain perception by means of associated variables such as catastrophizing, pain acceptance, and quality of life in people with chronic pain.

The study was based on the hypothesis that participants assigned to the intervention group will exhibit less catastrophizing and emotional distress, more acceptance of pain, and improved ability to perform activities of daily living according to self-management values. Moreover, they will experience a better quality of life and fewer symptoms, pain intensity, anxiety, and depression. These outcomes will be measured after completion of the intervention period and at the 3-month follow-up and compared with a control group in line with the recommended outcomes in chronic pain research [36].

## Methods

### Study Design

**Figure 1:**
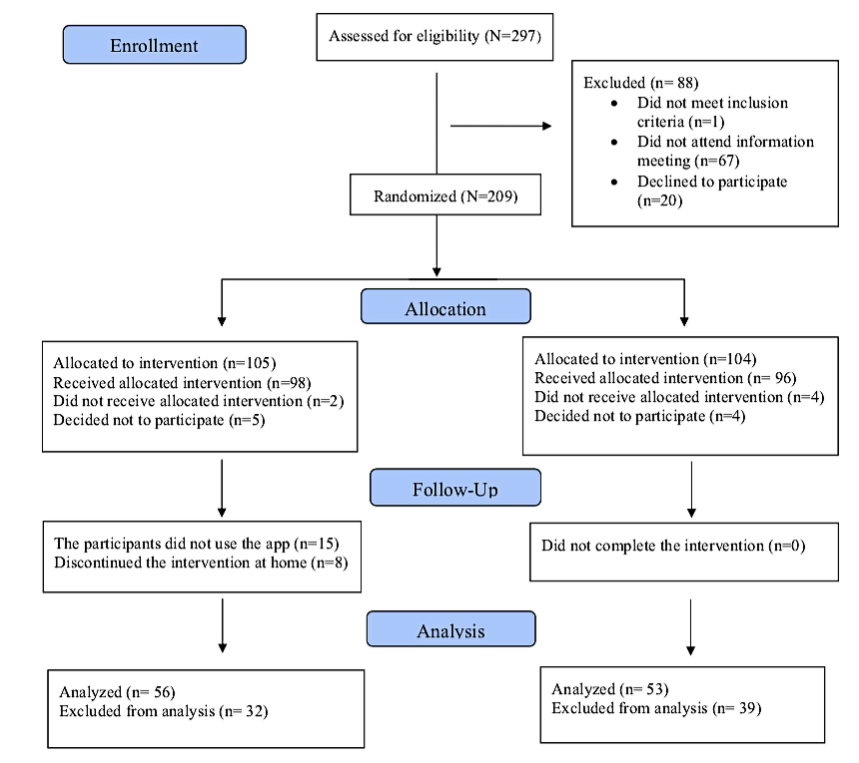
Flowchart of the recruitment process.

A randomized controlled clinical trial was performed using parallel treatment groups [31,37]. Block randomization was used to ensure a similar number of participants in each group and in the intervention phase (Figure 1).

### Mobile App–Based Multimodal Treatment

To develop the contents of the multimodal treatment for the mobile device app, a systematic literature review was carried out in a first phase following the Scottish Intercollegiate Guideline Network.

As part of a broader research program aimed at improving pain management, we first located articles that described the basic concepts of multi-professional treatments for chronic pain, provided recommendations, and included published evidence for the selected topics of interest. The purpose was to identify the characteristics of pharmacological and nonpharmacological interventions for people affected by chronic pain in different settings by means of a bibliographic search and reading of the literature, a synthesis of the results, and an assessment of the evidence. In the second part of our study, a participatory approach was used to prioritize self-management recommendations for chronic pain selected from the reviewed literature. Specifically, we designed a multimodal intervention protocol combining physical exercise, psychoeducational therapy, health assets, and pharmacological treatment that was delivered using a mobile device. The effectiveness of the intervention was subsequently evaluated by means of a clinical trial [38].

The interventions delivered in the mobile app include ACT and mindfulness exercises to promote greater pain acceptance, reduce the aversive component associated with pain, and help patients dispassionately recognize and observe both pain and related thoughts and emotions. Another group of activities aims to raise awareness of an individual’s own values through a series of activities to recover a meaningful life project [22,23,39,40]. The exercise section provides tools and resources for patients to improve their physical, mental, and emotional well-being. The activities in this section include empowerment, stretching, relaxation, walking, and low-intensity exercises to help patients acquire good habits and learn about alternatives to improve their day-to-day life [5]. The activities in the pharmacological section aim to help patients better understand medications that reduce the intensity of pain. For each medication, the most common side effects and characteristics related to pain relief are described as well as which drugs are best suited to the patient’s current health state, the risks of taking more than the recommended dose of medication, and how to identify warning signs [41-43]. The activities in the health assets section are designed to improve patients’ self-esteem and health by having them identify the individual, physical, institutional, associational, economic, and cultural assets and resources available in their community to help them cope better with situations of vulnerability and stress [44,45].

### Development of the Mobile App

Our research team developed an app called NO+Dolor (NO+Pain) that contains an Android or iOS user interface. The app includes links to several multimedia resources (mainly audios and videos) and was designed based on game dynamics (gamification) to improve users’ concentration, attention, and motivation. Indications on how to use the app and correctly perform the measures are provided in the instructions section of the app. Regarding the technical characteristics of the app, the information technology part required the design of a relational database implemented using MySQL Community Server 5.6. On the server side, a Java 1.8 communications application programming interface was implemented with representational state transfer architecture between the patients’ mobile apps and the database using a client-server pattern. On the client side, hybrid mobile apps were implemented for Android and iOS operating systems. Apache Cordova 9.0 and jQuery Mobile 1.4.5 frameworks were integrated into the app, and HTML5 and CSS3 technologies were used for the presentation layer [46].

### Participants

The study population comprised residents of municipalities belonging to the Cordoba South Health District of the Andalusian Health Service, Spain, who were registered in the health service user database. Patients were recruited from each of the district’s 11 primary care centers by two collaborators: a nurse and a physician with experience in the follow-up of patients with chronic pain.

The sample was drawn from a database using Diraya electronic medical records. The inclusion criterion was being attended by primary care physicians and nurses. The database search was carried out on June 30, 2019, and identified 297 patients diagnosed with chronic musculoskeletal pain, of which 205 (69%) were women and 92 (31%) were men. Accepting an *α* error of .05 and a *β* error of .2 (statistical power of 80%) in a 2-tailed test and estimating 15% loss to follow-up, 296 participants were required to detect a statistically significant difference between 2 means at 3 points in our outcome variable with an estimated SD of 12.

All participants were under pharmacological treatment for chronic musculoskeletal pain (analgesics, anti-inflammatory drugs, antidepressants, anticonvulsants, or opioids) as previously indicated by their primary care physician and were asked to sign an informed consent form. The inclusion and exclusion criteria for the participants in the study are presented in Textbox 1.

**Inclusion and exclusion criteria.**

**Inclusion criteria**

- Patients aged ≥18 years with pain in any location
- Pain with a duration of ≥3 months
- Pain with an intensity of ≥4 on the Visual Numerical Scale
- Presenting one of the following characteristics: continuous pain or intermittent pain ≥5 days a week
- Able to use a smartphone
- Not participating in another research project

**Exclusion criteria**

- Cancer-related or postsurgical pain, patients with palliative care, and pediatric population
- Patients with acute pain (duration <3 months)
- History of brain injury
- Inability to complete the study forms because of mental disability or language barrier

### Ethics Approval

This study was approved by the Cordoba Research Ethics Committee of the Andalusian Public Health System (CIF G-14825277; protocol code PI-0447-2017). Informed consent was approved by the Cordoba Research Ethics Committee and completed by the participants. This study is registered at ClinicalTrials.gov (NCT04509154).

### Randomization and Blinding

Block randomization with a block size of 4 was used. The only stratification criterion was the reference health center of the patients. An automated recruitment form hosted on the REDCap (Research Electronic Data Capture; Vanderbilt University) platform of the Maimonides Biomedical Research Institute of Cordoba was used to randomize the patients by simply clicking a button. The data were transferred and recorded in an electronic notebook using the Data Entry Manager system. The statistician (Ipek Guler Caamaño and Juan Antonio Marín Sanz), principal investigator (YM-M), and coinvestigators (AJS-G, SC-F, and MIB-P) of the study who evaluated the results were not involved in patient recruitment and were blinded to the group assignment. A total of 22 recruiters from the 11 primary care health centers of the Cordoba South Health District recruited the patients in 2019. They were also responsible for randomizing the patients (by clicking a button on the automated recruitment form) and were not blinded.

### Treatment Procedures

All patients received a written invitation from their primary care physician or nurse to participate in the study. Two 8-hour face-to-face sessions were held at their reference health center led by a nurse and a primary care physician with experience in the follow-up of patients with chronic pain. At the group meeting, patients who agreed to participate voluntarily in the study were informed that they would receive instructions by email on how to download the mobile app with the treatment contents. The patients were also informed that, if they were selected to participate in the intervention group, the treatment would last from 6 to 8 weeks. The control group would only have access to the *Find out more* section of the app, which contained audiovisual materials for pain management from a self-help approach, such as information on the origin of chronic pain and advice for pain treatment and relaxation exercises.

The intervention group received the treatment via their smartphones for a period of 6 weeks after completing both face-to-face sessions. Pain intensity was measured daily on an 11-point numerical rating scale when the participants accessed the app.

To assess the treatments, self-reported questionnaires were sent to the participants by email and collected at three time points: upon admission to the program (T1), at week 6 of the intervention (T2), and at 3 months after the intervention (T3). The participants completed all 3 questionnaires at home and returned them by email.

### Smartphone-Based Intervention

The intervention consisted of the implementation of a program of standard, interactive psychosocial therapy activities. The pain management app enables automatic monitoring, skill training, social support, education, goal setting, and achievement of four components: psychological wellness, exercise, pharmacological treatment, and health assets. Each week, the participants received 3 activities for each of the aforementioned components via the NO+Dolor app until completing the 6 weeks of treatment. All the activities were designed to be performed weekly except for the walking challenge, which was performed daily. The first time the participants completed an activity, they were awarded a star. The participants could perform the proposed activity as many times as they liked, but no more rewards were given until the following week. The more activities they completed, the more stars they were awarded, and the higher the percentage of goals reached by the patients each week (Multimedia Appendix 1). The app also had a *Consultation* section with a contact form where the participants could send any questions or comments. The form was then sent by email to the researchers so that they could respond to the inquiries.

### Assessment Measures

The Spanish adaptation [47,48] of the Pain Catastrophizing Scale (PCS) [49] was used to measure the main outcome variable of the study [50]. The total score on the PCS is calculated by summing the responses to the 13 items and ranges from 0 to 52. The PCS subscales comprise three dimensions: (1) rumination, scored from 0 to 16 (difficulty inhibiting repetitive pain-related thoughts and inability to seek solutions); (2) magnification, scored from 0 to 12 (tendency to exaggerate distressing situations and negative aspects of pain and perception of oneself as unable to control pain); and (3) hopelessness or helplessness, scored from 0 to 24 (inability to cope effectively with pain). Higher scores indicate higher levels of catastrophizing. A score of ≥30 is considered a cutoff point for clinically significant catastrophizing levels. The Spanish version of the PCS has been shown to have adequate internal consistency (Cronbach *α*=.79), convergent validity and classificatory value, test-retest reliability (intraclass correlation coefficient=0.84), and sensitivity to change in size (effect size>2).

The Spanish adaptation [51] of the Chronic Pain Acceptance Questionnaire (CPAQ) [21] was used to measure engagement in life activities despite pain; willingness to experience pain without trying to control, change, or avoid it; ability to recognize the chronicity of pain; and the need to avoid or control pain. The CPAQ is a 20-item self‑report questionnaire that rates pain acceptance on a 7-point Likert-type scale ranging from 0 (*never true*) to 6 (*always true*). The maximum possible score on the CPAQ is 120, with higher scores indicating higher pain acceptance. Initial studies on the acceptance and adaptation of the CPAQ have shown adequate internal consistency and expected correlations with measures of physical functioning and psychological distress. Subsequent studies have evaluated the content and dimensions of the questionnaire and identified two factors: activity engagement (Cronbach *α*=.82) and pain willingness (Cronbach *α*=.78) [21].

The Spanish adaptation of the EQ-5D [52] was used to measure health-related quality of life. This version can be used both in relatively healthy individuals (the general population) and in groups of patients with different conditions. Individuals assess their own health state first by level of severity in different dimensions (descriptive system) and then on the more general EuroQol Visual Analogue Scale (EQ-VAS) of 0 to 100 (*worst imaginable health state* and *best imaginable health state*, respectively). A third component of the EQ-5D is the social values index obtained for each health state generated by the instrument, which describes respondents’ health state according to five dimensions: mobility, self-management, usual activities, pain or discomfort, and anxiety or depression. Regarding the instrument’s psychometric properties, the test-retest reliability ranges from 0.86 to 0.90, and numerous studies have demonstrated its validity and sensitivity to change [53]. We included a question on subjective global improvement rated by the EQ-VAS from 0 to 100: *We would like you to indicate on this scale how good or bad your health state is today*. Pain intensity was measured using an 11-point numerical rating scale ranging from 0 (*no pain*) to 10 (*pain as bad as you can imagine*). The format of this rating was established in the latest Initiative on Methods, Measurement, and Pain Assessment in Clinical Trials recommendations on core outcome measures for chronic pain clinical trials [36].

### Data Analysis

A descriptive analysis was performed for the quantitative variables with mean and SD and for the qualitative variables with recounts (n) and proportions (%). Goodness-of-fit to a normal distribution was determined using the Shapiro-Wilk test, and homogeneity of variance was assessed using the Levene test. The quantitative variables of the treatment and control groups were compared using the Mann-Whitney *U* test, and the Pearson chi-square test was used to compare the qualitative variables and the Fisher-Freeman-Halton descriptive analysis. In addition, the variations between the different phases were assessed using the percentage change rescaled with log base 2. The association between the quantitative variables was determined using bivariate (Pearson linear correlation coefficient or Spearman *ρ*) and partial correlations controlling for the variables age and sex.

A linear mixed effects model [54,55] was subsequently used to assess changes over time for the repeated measurements of the pain questionnaire scores at 3 time points between the control and treatment groups. Linear mixed effects models account for variability between participants and between repeated measurements in the same participant simultaneously. To obtain different trajectories for each group (experimental vs control) over time, we included the intercept and slope effect as random effects and time, group, and the interaction term (group×time) as fixed effects. The variance-covariance structure was fixed to an unstructured matrix, and the random effects and error terms were assumed to have a normal distribution. Furthermore, the Cohen *d* was calculated based on the results of the linear mixed model. The R project *nlme* package (version 3.5.0; R Foundation for Statistical Computing) was used to estimate all the regression models. The established level of statistical significance was *P*<.05.

## Results

### Participants

A total of 297 participants (n=205, 69% women and n=92, 31% men) were initially invited to participate in the study. Of these 297 participants, 1 (0.3%) was excluded for not meeting the inclusion criteria, 67 (22.6%) were excluded because they did not attend the meeting, and 20 (6.7%) were excluded because they declined to participate. A total of 209 participants were randomized into 1 of the 2 study arms. After randomization, 2.4% (5/209) of the participants were excluded because they declined to participate, dropped out before the intervention, or did not know how to use the technologies. In the intervention group, 15% (15/98) of the patients did not complete the intervention. The analysis was performed on patients who completed all 3 questionnaires at baseline, upon completion of the intervention, and 3 months after the intervention (Figure 1).

Table 1 shows the demographic data and baseline characteristics of the sample by group. The outcome variables showed measures for PCS (*P*=.20), CPAQ (*P*=.07), and EQ-5D (*P*=.26) at the beginning of the intervention (baseline), in which participants in both groups did not differ in pain catastrophizing, quality of life, or pain acceptance. No differences were found in sociodemographic variables or use of medications (Table 1).

**Table 1:** Characteristics of the sample (N=194).

Characteristic			Intervention group (n=98)		Control group (n=96)		*P* value	
Age (years), mean (SD)			51.2 (11.2)		50.3 (10.2)		.99^a^	
**Gender, n (%)**								.99
	Female	78 (80)		77 (80)				
	Male	20 (20)		19 (20)				
**Marital status, n (%)**								.59
	Married	81 (83)		78 (81)				
	Divorced	6 (6)		8 (8)				
	Single	6 (6)		6 (6)				
	Widowed	2 (2)		4 (4)				
**Employment status, n (%)**								.27
	Unemployed	17 (17)		15 (16)				
	Employed full-time	27 (28)		29 (30)				
	Employed part-time	13 (13)		3 (3)				
	Disability	11 (11)		12 (13)				
	Home care	22 (22)		28 (29)				
	Other combination of the aforementioned characteristics or unknown	8 (8)		9 (9)				
**Level of education, n (%)**								.16
	No schooling	13 (13)		7 (7)				
	8 to 10 years (elementary)	41 (42)		54 (56)				
	10 to 12 years (high school)	36 (37)		26 (27)				
	>12 years (higher education)	8 (8)		9 (9)				
**Taking medications, n (%)**								.68
	Yes	96 (98)		92 (96)				
	No	2 (2)		4 (4)				
**Annual income, € (US $), n (%)**								.45
	<10,000 (10,857)	11 (11)		12 (26)				
	10,000 to 20,000 (10,857 to 21,714)	73 (74)		74 (77)				
	>20,000 (21,714)	14 (14)		10 (5)				
**EQ-5D and pain, n (%)**			56 (100)		53 (100)		.34	
	Moderate pain	34 (61)		27 (51)				
	Extreme pain	22 (39)		26 (49)				
**EQ-5D and anxiety and depression, n (%)**			56 (100)		52 (100)		.90	
	I am not anxious or depressed	13 (23)		13 (25)				
	I am moderately anxious or depressed	32 (57)		30 (58)				
	I am extremely anxious or depressed	11 (20)		9 (17)				
PCS^b^, mean (SD)			29.86 (13.27)^c^		27.7 (12.77)^d^		.20	
CPAQ^e^, mean (SD)			64.16 (18.89)^d^		66.77 (18.4)^f^		.07	
EQ-5D^g^, mean (SD)			0.45 (0.19)^h^		0.43 (0.21)^i^		.26	
EQ-VAS^j^, mean (SD)			48.22 (18.74)^k^		44.23 (23.49)^l^		.16	

^a^n=194.

^b^PCS: Pain Catastrophizing Scale (score 0-52).

^c^n=55.

^d^n=54.

^e^CPAQ: Chronic Pain Acceptance Questionnaire (score 0-120).

^f^n=52.

^g^EQ-5D score −0.654 to 1.

^h^n=53.

^i^n=51.

^j^EQ-VAS: EuroQol Visual Analogue Scale (0-100).

^k^n=50.

^l^n=47.

### Primary Outcome: Catastrophizing

#### Overview

The descriptive results for the differences between the control and intervention groups regarding measures of catastrophizing at baseline, upon completion of the treatment, and during follow-up are presented in Table 2. The between-group variations over time for the primary outcome variables are presented in Table 3.

**Table 2:** Median, mean, SD, and differences between groups for the primary outcome measure at admission to the program (T1), immediately after treatment (T2), and at the 3-month follow-up (T3) for completers.

Primary outcome measure (scale)		T1^a^					T2^a^							T3^a^				
		Median (IQR)	Mean (SD)	*P* value		Median (IQR)		Mean (SD)		*P* value		Median (IQR)			Mean (SD)		*P* value	
**PCS^b^**					.20						.001							.09
	Control	26.5 (17.25-38.5)	27.7 (12.77)^c^			32.5 (23.75-42.0)		31.82 (12.06)^d^				31.0 (22.5-43.0)			31.41 (12.61)^e^			
	Intervention	29.0 (22.0-39.0)	29.86 (13.27)^f^			19.5 (14.25-28.25)		20.86 (11.25)^g^				27.0 (17.0-35.0)			25.78 (14.12)^h^			
**Helplessness**					.31						.001							.07
	Control	12.5 (8.0-16.25)	12.48 (5.8)^i^			14.0 (10.5-19.0)		14.22 (5.82)^e^				14.0 (9.75-18.75)			13.65 (5.75)^j^			
	Intervention	14.0 (8.25-18.0)	13.09 (6.45)^c^			8.5 (5.25-12.0)		8.91 (5.38)^g^				10.0 (6.0-17.0)			11.09 (6.48)^g^			
**Rumination**					.14						.004							.12
	Control	10.0 (6.0-12.0)	9.19 (4.16)^k^			7.0 (4.0-8.25)		6.32 (2.99)^d^				6.0 (4.0-9.0)			6.28 (2.84)^l^			
	Intervention	10.0 (7.5-14.0)	10.11 (4.11)^m^			4.0 (2.0-5.0)		4.05 (2.4)^n^				5.0 (3.0-7.0)			5.24 (3.16)^n^			
**Magnification**					.20						.007							.12
	Control	6.5 (4.0-8.75)	6.57 (3.17)^c^			13.0 (8.75-14.25)		11.29 (4.28)^d^				12.0 (7.5-15.5)			11.33 (4.56)^e^			
	Intervention	7.0 (4.25-9.75)	7.06 (3.22)^c^			8.0 (6.0-12.0)		8.29 (4.14)^n^				10.5 (5.75-12.0)			9.55 (5.04)^g^			

^a^At admission to the program (T1), at the end of the intervention at 6 weeks (T2), and at the 3-month follow-up (T3).

^b^PCS: Pain Catastrophizing Scale.

^c^n=54.

^d^n=28.

^e^n=27.

^f^n=56.

^g^n=22.

^h^n=23.

^i^n=52.

^j^n=26.

^k^n=53.

^l^n=25.

^m^n=55.

^n^n=21.

**Table 3:** Between-group differences and changes over time for the primary outcome measure and Pain Catastrophizing Scale (PCS) subscales.

Primary outcome measure (scale)			T2–T1^a^								T3–T2^a^										T3–T1^a^						
			Median (IQR)		Mean (SD)		*P* value			Median (IQR)			Mean (SD)			*P* value			Median (IQR)				Mean (SD)			*P* value	
**PCS**								<.001									.24										.006
	Control	0.05 (−0.11 to 0.73)		0.29 (1.0)^b^					−0.03 (−0.19 to 0.21)			−0.07 (0.32)^c^						0.1 (−0.14 to 0.36)				0.21 (1.13)^d^					
	Intervention	−0.5 (−1.0 to −0.14)		−0.67 (0.72)^e^					0.0 (−0.27 to 0.58)			0.02 (0.65)^f^						−0.15 (−1.0 to 0.08)				−0.59 (0.91)^e^					
**Helplessness**								.002									.21										.007
	Control	0.0 (−0.23 to 0.4)		0.1 (0.84)^g^					−0.07 (−0.41 to 0.04)			−0.11 (0.33)^h^						0.0 (−0.32 to 0.48)				0.01 (0.88)^i^					
	Intervention	−0.79 (−1.14 to −0.07)		−0.72 (0.78)^j^					0.0 (−0.35 to 0.44)			0.11 (0.8)^f^						−0.33 (−1.06 to −0.08)				−0.65 (0.92)^j^					
**Rumination**								<.001									.48										.049
	Control	−0.58 (−0.83 to 0.0)		−0.53 (0.89)^k^					0.0 (0.0 to 0.39)			0.29 (0.86)^h^						−0.71 (−1.0 to −0.26)				−0.59 (0.96)^i^					
	Intervention	−1.42 (−2.27 to −0.79)		−1.59 (0.95)^l^					0.0 (0.0 to 0.57)			0.22 (0.55)^h^						−0.85 (−1.82 to −0.58)				−1.23 (0.84)^l^					
**Magnification**								.14									.41										.03
	Control	0.56 (0.32 to 0.97)		0.77 (1.0)^b^					0.12 (−0.14 to 0.22)			−0.02 (0.48)^c^						0.68 (0.49 to 1.25)				0.86 (1.02)^d^					
	Intervention	0.42 (0.29 to 0.66)		0.2 (1.02)^l^					0.0 (−0.13 to 0.46)			−0.03 (0.83)^f^						0.58 (−0.43 to 0.68)				0.1 (1.16)^j^					

^a^At admission to the program (T1), at the end of the intervention at 6 weeks (T2), and at the 3-month follow-up (T3).

^b^n=26.

^c^n=16.

^d^n=27.

^e^n=21.

^f^n=17.

^g^n=23.

^h^n=15.

^i^n=24.

^j^n=20.

^k^n=25.

^l^n=19.

#### Between-Group Effects

Immediately following the treatment (T2), statistically significant improvements were observed in the intervention group for catastrophizing (20.86 vs 31.82; *P*=.001) and the catastrophizing subscales of helplessness (8.91 vs 14.22; *P*=.001), rumination (4.05 vs 6.32; *P*=.004), and magnification (8.29 vs 11.29; *P*=.007). However, at 3 months of follow-up, the mean remained lower (25.78 vs 31.41; *P*=.09), although it was not statistically significant (Table 2).

#### Within-Group Effects

Positive effects were observed at the different treatment times according to the percentage change rescaled by log base 2. Specifically, positive effects were found for the intervention group (T2–T1) in catastrophizing between the baseline and posttreatment phases (*P*<.001) and in the subscales of helplessness (−0.72 vs 0.1; *P*=.002) and rumination (−1.59 vs −0.53; *P*<.001), although no significant changes were found for magnification (0.2 vs 0.77; *P*=.14). Significant results were also found for catastrophizing at the 3-month follow-up with respect to the baseline (−0.59 vs 0.2; *P*=.006) and the subscales of helplessness (−0.65 vs 0.01; *P*=.07), rumination (−1.23 vs −0.59; *P*=.04), and magnification (0.1 vs 0.86; *P*=.02), all of which improved 3 months after completing treatment (Table 3).

We also measured changes in the pain questionnaire scores between the control and treatment groups at the 3 time points by applying a linear mixed effects model. Statistical differences were found only between the 2 groups for changes in the PCS score over time. In addition, the interaction effect between time and the intervention group was −6.47 (*P*=.001), thus indicating a significant decrease in PCS scores over time in the intervention group compared with the control group (Table 4).

**Table 4:** Changes between groups in Pain Catastrophizing Scale scores over time.

Coefficient	Values, mean (SD)	*P* value
Intercept	25.747 (2.572)	<.001
Time	2.479 (1.336)	.07
Intervention (reference: control)	7.528 (3.669)	.04
Time^a^ intervention (reference: time control)	−6.476 (1.991)	.001

^a^Pain Catastrophizing Scale scores over time in the intervention group compared with the control group.

### Secondary Outcomes

#### Overview

Table 5 shows the results for the variables of acceptance (CPAQ), quality of life (EQ-5D), and overall health state (EQ-VAS), whereas Table 6 shows variations over time for the secondary outcomes.

**Table 5:** Mean and SD for the secondary outcomes at admission (T1), immediately after treatment (T2), and 3 months after the intervention period (T3) for completers.

Secondary outcome measure (scale)		T1^a^					T2^a^						T3^a^				
		Median (IQR)	Mean (SD)	*P* value		Median (IQR)		Mean (SD)		*P* value		Median (IQR)		Mean (SD)		*P* value	
**CPAQ^b^**					.07						.14						.47
	Control	68.0 (58.0-78.0)	66.77 (18.4)^c^			65.5 (52.75-72.5)		63.82 (12.47)^d^				65.0 (54.5-71.5)		65.67 (16.06)^e^			
	Intervention	63.0 (53.75-71.0)	64.16 (18.89)^f^			67.0 (59.0-78.0)		68.23 (14.43)^g^				62.0 (54.5-73.0)		64.48 (21.76)^h^			
**EQ-5D**					.27						.008						.30
	Control	0.46 (0.22-0.59)	0.43 (0.21)^c^			0.36 (0.22-0.56)		0.41 (0.22)^d^				0.37 (0.22-0.52)		0.39 (0.19)^e^			
	Intervention	0.48 (0.23-0.59)	0.45 (0.19)^f^			0.56 (0.47-0.72)		0.55 (0.17)^g^				0.41 (0.22-0.59)		0.43 (0.2)^h^			
**EQ-VAS^i^**					.16						.02						.03
	Control	44.0 (21.5-54.0)	44.23 (23.49)^j^			35.0 (25.25-56.0)		38.68 (19.58)^g^				30.0 (25.0-51.0)		36.96 (20.15)			
	Intervention	47.0 (33.5-60.0)	48.22 (18.74)^k^			53.0 (38.0-66.0)		52.68 (18.27)^l^				50.0 (31.5-75.0)		51.05 (25.73)^l^			

^a^At admission to the program (T1), at the end of the intervention at 6 to 7 weeks (T2), and at the 3-month follow-up (T3).

^b^CPAQ: Chronic Pain Acceptance Questionnaire.

^c^n=53.

^d^n=28.

^e^n=27.

^f^n=56.

^g^n=22.

^h^n=23.

^i^EQ-VAS: EuroQol Visual Analogue Scale.

^j^n=47.

^k^n=50.

^l^n=19.

**Table 6:** Within-group differences and variations over time for the secondary outcomes.

Secondary outcome measure (scale)		T2–T1^a^					T3–T2^a^							T3–T1^a^				
		Median (IQR)	Mean (SD)	*P* value		Median (IQR)			Mean (SD)		*P* value		Median (IQR)		Mean (SD)		*P* value	
**CPAQ^b^**					.001							.14						.30
	Control	−0.16 (−0.27 to 0.1)	0.05 (0.9)^c^			0.02 (−0.12 to 0.25)			0.08 (0.28)^d^				−0.08 (−0.24 to 0.09)		0.12 (0.92)^e^			
	Intervention	0.22 (0.05 to 0.39)	0.38 (0.86)^f^			−0.1 (−0.24 to 0.16)			−0.22 (0.95)^g^				0.0 (−0.18 to 0.25)		−0.1 (0.86)^f^			
**EQ-5D**					.002							.17						.48
	Control	−0.12 (−0.53 to 0.08)	−0.1 (0.69)^c^			0.0 (−0.03 to 0.22)			0.04 (0.34)^d^				0.0 (−0.4 to 0.45)		0.12 (0.69)^e^			
	Intervention	0.31 (0.0 to 0.92)	0.43 (0.66)^f^			0.0 (−0.31 to 0.13)			−0.09 (0.61)^g^				0.0 (−0.1 to 0.35)		0.08 (0.75)^f^			
**EQ-VAS^h^**					.13							.49						.34
	Control	0.14 (−0.77 to 0.49)	−0.27 (1.0)			−0.05 (−0.28 to 0.17)			−0.08 (0.31)				0.18 (−0.34 to 0.83)		0.18 (1.22)^i^			
	Intervention	0.28 (−0.12 to 0.42)	0.25 (0.52)			−0.14 (−0.37 to 0.24)			−0.05 (0.4)				0.33 (−0.26 to 0.95)		0.18 (0.93)^d^			

^a^At admission to the program (T1), at the end of the intervention at 6 weeks (T2), and at the 3-month follow-up (T3).

^b^CPAQ: Chronic Pain Acceptance Questionnaire.

^c^n=26.

^d^n=16.

^e^n=27.

^f^n=21.

^g^n=17.

^h^EQ-VAS: EuroQol Visual Analogue Scale.

^i^n=24.

#### Between-Group Effects

With regard to pain acceptance (CPAQ), no significant differences were found between the 2 groups after treatment (68.23 vs 63.82; *P*=.14) or at 3 months following the intervention (64.48 vs 65.67; *P*=.47).

In terms of quality of life (EQ-5D), the intervention group showed significant improvement at the end of treatment (0.55 vs 0.41; *P*=.008), although these differences were not maintained after the 3-month follow-up (0.43 vs 0.39; *P*=.30).

The assessment of overall health state (EQ-VAS) registered in the daily records showed significant improvements in the intervention group compared with the control group (52.68 vs 38.68; *P*=.02) at the end of treatment, and these differences were maintained over time (51.05 vs 39.96; *P*=.02; Table 5).

#### Within-Group Effects

Regarding variations between the different phases, a positive effect was observed immediately following the intervention (T2–T1) in both acceptance (0.38 vs 0.05; *P*=.001) and quality of life (0.43 vs −0.01; *P*=.002), but the positive effect on overall satisfaction with health was not maintained (0.25 vs −0.27; *P*=.13).

No significant differences were found during follow-up for CPAQ (T3–T2: −0.22 vs 0.08 and *P*=.14; T3–T1: −0.1 vs 0.12 and *P*=.30), EQ-5D (T3–T2: −0.09 vs 0.04 and *P*=.17; T3–T1: 0.08 vs 0.12 and *P*=.48), or overall health state (T3–T2: −0.05 vs −0.08 and *P*=.49; T3–T1: 0.18 vs 0.18 and *P*=.34; Table 6).

Table 7 shows the Fisher-Freeman-Halton descriptive analysis of the proportion of participants with clinically significant improvement immediately after treatment according to the EQ-5D subscale. In the intervention group, clinical improvement in pain intensity ranged from 37% in moderate pain to 22.7% in severe pain (*P*=.04). The exact test results showed that a significantly higher proportion of participants who received the multimodal treatment improved in the mobility subscale (*P*=.04) and activities of daily living such as going to work, leisure time, and family activities (*P*=.045) immediately after receiving treatment. These improvements were not significant 3 months after the intervention.

**Table 7:** Proportion of patients to the EQ-5D subscale per time and group at admission (T1), immediately after treatment (T2), and 3 months after the intervention period (T3).

EQ-5D secondary outcome measure		T1					T2							T3				
		Intervention, n (%)	Control, n (%)	Overall *P* value		Intervention, n (%)		Control, n (%)		Overall *P* value		Intervention, n (%)			Control, n (%)		Overall *P* value	
**Pain**					.34						.004							.08
	Moderate pain (VAS^a^=4-7)	34 (61)	27 (51)			16 (37)		10 (78)				13 (34)			9 (53)			
	A lot of pain (VAS≥8)	22 (39)	26 (49)			2 (23)		17 (64)				10 (46)			17 (67)			
**Anxiety and depression**					.90						.27							.12
	I am not anxious or depressed	13 (23)	13 (25)			7 (32)		8 (29)				3 (13)			8 (30)			
	I am moderately anxious or depressed	32 (57)	30 (58)			13 (59)		12 (43)				16 (67)			14 (52)			
	I am very anxious or depressed	11 (20)	9 (17)			1 (9)		7 (29)				4 (21)			4 (19)			
**Mobility**					.14						.04							.07
	I have no problem walking	9 (41)	10 (36)			12 (21)		13 (25)				7 (29)			8 (30)			
	I have some trouble walking	43 (77)	39 (74)			13 (59)		18 (64)				16 (67)			19 (70)			
	I have to be in bed	1 (2)	1 (2)			N/A^b^		N/A				1 (4)			N/A			
**Personal care**					.92						.77							.63
	I have no problems with self-care	33 (60)	31 (59)			14 (65)		15 (56)				12 (50)			13 (50)			
	I have some problems washing or dressing	22 (40)	21 (40)			8 (36)		12 (44)				12 (50)			14 (50)			
	I am unable to wash or dress	N/A	1 (2)			N/A		N/A				N/A			N/A			
**Everyday activities (work, study, household chores, free time, and family activities)**					.14						.045							.86
	I have no problem performing my daily activities	5 (9)	9 (17)			5 (24)		4 (14)				2 (8)			1 (4)			
	I have some trouble performing my daily activities	49 (88)	38 (72)			16 (76)		20 (71)				20 (83)			23 (85)			
	I am unable to perform my daily activities	2 (4)	6 (11)			N/A		4 (14)				2 (8)			3 (11)			

^a^VAS: Visual Analogue Scale.

^b^N/A: not applicable.

## Discussion

### Principal Findings

We performed a small randomized controlled clinical trial with a sample of mostly women (205/297, 69%). Randomization of both the sociodemographic characteristics of the study population and the study variables was homogeneous.

The results of the study clearly show that the main variable, catastrophizing, had a significant positive effect on the intervention group compared with the control group after the intervention and at the 3-month follow-up. This could be due to changes in cognition or maladaptive behavior that modified erroneous beliefs and decreased catastrophic thoughts. It should be noted that previous research has considered the beneficial effect of multimodal treatments in decreasing catastrophizing and fear. Thus, these results are in accordance with the more pronounced effect found in the intervention group [55].

These results are also in line with the findings of studies on the effects of monotherapies using ACT [56], mindfulness [57], physical activity, pharmacological therapy, and the health asset approach. Analysis of the results showed that the intervention group improved in catastrophizing, psychological flexibility, movement avoidance, pain interference in daily life, pain intensity, and quality of life [31,58-62].

The analysis showed that the intervention group improved in both perceived quality of life and pain acceptance after treatment. This finding indicates that patients who accept pain better exhibit greater psychological flexibility (ie, less activity avoidance), less psychological distress, and less disability. Moreover, we found that patients with higher pain acceptance (psychological flexibility; *P*=.001) reported a better quality of life (*P*=.002). This is in agreement with other studies that have shown that pain acceptance is a good predictor of a better quality of life [20,21,61].

However, the results were inconclusive for these last 2 variables. Specifically, although the effect sizes of quality of life and pain acceptance were significant from before treatment to after treatment in the intervention group, no changes were observed 3 months after the treatment. This lack of significance may be explained by the fact that perceived quality of life is a multidimensional phenomenon, and some indicators may therefore have had a greater influence on this variable, such as the low educational level and low annual income of the sample. These results are consistent with those of other studies suggesting that low educational level and low income are associated with poorer perceived health [63].

With regard to the main aim of this work, we hypothesized that the participants assigned to the intervention group would experience fewer catastrophizing thoughts and emotional distress and more acceptance of pain and improve their ability to perform activities of daily living according to self-management values. The results are encouraging as the effects of the treatment were largely maintained over time and reduced catastrophizing and the three dimensions of the PCS (helplessness, rumination, and magnification). This may suggest that a combination of these interventions promotes skills that result in behavior changes, at least in the medium term.

A priori, this relationship could be explained by the additive effect of the combined components of psychoeducational therapy, exercise, pharmacological treatment, and health assets. It may also be explained by the fear avoidance model in that exercise contributes to the reconceptualization of pain and reduces catastrophic thinking and the threat value of pain related to functional limitations [55]. Similarly, exercise may help divert attention away from rumination because of its attentional demands and mood effects, whereas the use of exercise as a self-management tool could increase self-efficacy and thereby reduce feelings of helplessness.

Physical activity likely helped the participants in our study learn about activities such as daily walking and low-intensity stretching to improve their physical, mental, and emotional well-being as well as acquire new habits and find alternatives to improve their quality of life [64]. These results are in line with recent studies and meta-analyses that show that intermittent or regular sessions of therapeutic exercise can reduce pain perception and sensitivity [65].

ACT and mindfulness were also a treatment goal in our study [60]. In the intervention group, both therapies had a positive effect on pain acceptance (psychological flexibility), improved health perception, and decreased levels of catastrophizing immediately after the intervention. This finding is in line with the available evidence suggesting that face-to-face or technology-based ACT is an effective self-management intervention for chronic pain and that it may be effective for the treatment of chronic pain [23,34,66].

Pain catastrophizing has been identified as a psychosocial factor that predicts adaptation to chronic pain and may contribute to its development and chronicity. In this regard, several studies [28,67] have examined the associations between pain catastrophizing and patient functioning and suggested that genetic and interpersonal factors, family history, pain duration, and comorbidities moderate pain and are likely to influence the strength of the association of the effects of catastrophizing on pain.

However, another explanation for the effect of our multimodal treatment may be related to patient-treatment matching. Specifically, broader-spectrum multimodal treatments have a greater likelihood of matching at least one treatment component to a patient’s strength or deficit [68].

The overall health state self-reported by the intervention group improved after the intervention, although this effect was not maintained over time. This may be explained by the fact that the participants perceived an improvement in their health state when performing the proposed weekly activities during the treatment period because of the effort and time invested, which is known as the Hawthorne phenomenon [69]. Motivation, or the effect of feeling observed and cared for, may also have played a role. It is important to remember that it takes time to modify lifestyles and habits and see the benefits of change.

Moreover, the descriptive analysis of the EQ-5D dimensions showed improvement in at least three of the five core outcome domains (pain intensity, mobility, and activities of daily living) compared with the control group. This finding corroborates the influence of attitudes and beliefs that may affect the development of passive coping mechanisms such as rest and medication versus the ability to adopt active strategies such as physical activity and pain acceptance [70].

### Strengths, Limitations, and Future Directions

This study has several strengths. First, we developed a multimodal program involving a variety of therapeutic activities that could be standardized and used in the future for other patients with chronic pain. An encouraging finding in this line was that the benefits of the treatment were largely maintained at follow-up, which may suggest that these interventions lead to the acquisition of skills that result in behavior change, at least in the medium term. Second, ICTs were used in combination with pharmacological and nonpharmacological therapeutic treatments in the outpatient setting to evaluate their impact on pain catastrophizing, pain acceptance, and quality of life. Third, the fact that the patients did the programmed activities in their natural environment is likely to promote self-efficacy, thus supporting the importance of the self-management component in interventions of this type [25].

One of the most important limitations of our study was the low response rate of the self-reported questionnaires sent via email. It should also be noted that the sample size was small. This may have affected the outcomes of the intervention and could explain the significant differences found between the groups. However, we cannot rule out the beneficial effect of the treatment on catastrophizing in the intervention group as a good prognosis of the disease and that, in the long term, these interventions (physical activity, psychoeducational therapies, pharmacological therapies, and health assets) are part of multi-professional treatments to achieve the desired effects. Nevertheless, as we used a multimodal therapy, we cannot really know the effect of each individual intervention on the outcomes, and more studies will be needed to determine the effects of specific interventions in patients with chronic pain. Therefore, caution must be exercised when extrapolating our findings to the general population.

With regard to treatment adherence [5,71], we cannot reach reliable conclusions. Although the electronic records of the participants’ access to and completion of the activities were not analyzed, we do know that 85% (83/98) of the participants completed the intervention program. This loss of participants may have occurred because the self-reported assessment was administered via email. Therefore, our treatment design could be improved by administering the questionnaire through the same mobile app, in interviews with the attending nurses, or by telephone follow-up.

According to our results and the available evidence, pharmacological treatments are most effective when they are part of an overall multidisciplinary pain management plan that also incorporates psychological, physical, and preventive components [8,72-74]. Clearly, patients with chronic pain should be informed and educated to enable them to make decisions about the most effective evidence-based strategy and ensure that their pain is treated and managed in the best possible manner.

A large number of studies have been conducted on ICT-based interventions to promote self-management in people with chronic pain [75-77], and there is evidence of high ICT acceptance and satisfaction [72,73]. Indeed, as technologies can assist and support people in their daily lives and at any time of the day [32], smartphones have become a very effective health care tool. In our smartphone app, we have selected evidence-based activities to address the various dimensions of chronic pain. The activities are easily reproducible in many environments and health care fields and can serve as complementary therapies for the comprehensive treatment of people with persistent pain. Nonetheless, it should be noted that self-management of pain is only effective when implemented from a multidisciplinary approach as treatment response is individual and there is no single approach that is beneficial for all patients with chronic pain. Therefore, for smartphone-based apps to be successful in promoting the self-management of chronic pain, we believe they should include self-monitoring, goal setting, skill training, social support, and educational components [78]. Moreover, many of these apps appear to have been developed without the involvement of patients and health care professionals and, to the best of our knowledge, few have been tested in randomized trials to evaluate their impact on health.

In future research, more attention should also be paid to the participants’ gender as this could have affected our findings. It is well-known that gender is strongly related to access to care and treatment response and that, although many patients who experience pain are women, many stigmas are associated with pain in men. Therefore, it is important that we gain a better understanding of the role of gender in health care access as well as gender biases in diagnoses, patient-professional interactions, and treatment.

Further lines of research could improve the efficacy of multimodal chronic pain interventions based on new technologies, such as the refinement of treatments, the identification of moderating factors that might influence psychosocial variables, and their association with treatment adherence. To evaluate which groups of patients are more competent to self-manage a technology-based multimodal intervention would have been ideal. In the same vein, it is also worth mentioning that, in our study, we did not specifically assess satisfaction in relation to the use of technologies, and this could be a promising line for future research.

In future research, it might be interesting to analyze data not included in this study, such as mobile sensor data using accelerometers, gyrometers, and other sensors to monitor engagement and assess the timing of the activities.

### Conclusions and Implications for Practice

The results of our study suggest that multidimensional treatments for adults with chronic pain improve catastrophizing, quality of life, and psychological flexibility immediately after treatment and that the effects are maintained for the primary outcome of catastrophizing for at least 3 months following treatment. This study has also shown that nonpharmacological treatments that include physical and psychoeducational therapy to promote active participation work well in combination with pharmacological strategies and that such interventions improve self-reliance in patients with chronic pain and help them cope constructively with pain.

The NO+Dolor app we have developed uses gamification to teach patients distraction methods and divert their attention away from pain as well as mindfulness techniques to improve pain acceptance. It also provides patients with a well-paced program of exercises and information on the proper use of medications to avoid side effects and helps them identify health assets to engage in pleasurable activities or find the resources they need. Moreover, the app-based mobile interventions we have presented here are flexible and self-directed, promote self-management in patients with chronic pain, and can be used to complement face-to-face pain treatments.

Preventive interventions for people with chronic pain designed from a salutogenic approach, are essential to promote well-being and prevent further decline in health throughout life.

## Appendix

Multimedia Appendix 1 Key contents of the intervention.

Multimedia Appendix 2 CONSORT-eHEALTH checklist (V 1.6.1).

## Abbreviations

ACT: acceptance and commitment therapy
CPAQ: Chronic Pain Acceptance Questionnaire
EQ-VAS: EuroQol Visual Analogue Scale
ICT: information and communication technology
PCS: Pain Catastrophizing Scale
REDCap: Research Electronic Data Capture

## Data Availability

The data supporting the reported results can be found in the electronic notebook of the Data Entry Manager of the Maimonides Biomedical Research Institute of Cordoba. The statistician, principal investigator, and collaborators who evaluated the results did not participate in patient recruitment.
