# Supplementary material for: Multimodal Chronic Pain Therapy for Adults via Smartphone: Randomized Controlled Clinical Trial
Source: J Med Internet Res. 2022 May 11;24(5):e36114. doi: 10.2196/36114 (PMC9133987; doi:10.2196/36114)
Supplement: Multimedia Appendix 1 [file jmir_v24i5e36114_app1.pdf]

Section	Week 1/Activities	Week 2/Activities	Week 3/Activities	Week 4/Activities	Week 5/Activities	Week 6/Activities
Exercise	Walking: Walk for at least 5 min	Walking: Walk for at least 10 min	Walking: Walk for at least 15 min	Walking: Walk for at least 20 min	Walking: Walk for at least 25 min	Walking: Walk for at least 30 minutes (min)
	Video: Cervical spine exercises for 3 min	Video: Spine exercises, Part 1 stretching and breathing (5 min)	Video: Strengthening of upper limbs, Part 1: Outdoor exercises with weights (5 min)	Video: Hamstrings, quadriceps and pyramidal muscle stretches (1 min)	Video: 'Yoga' sun salutation. Three times upon awakening (1.5 min)	Videos: Leg and torso stretches (2 min) and neck and torso stretches (2 min)
	Video: Leg toning exercises for 1.15 min	Video: Spine exercises, Part 2 (4 min)	Video: Strengthening of upper limbs, Part 2: Outdoor exercises with elastic bands (5 min)	Video: Back flexibility exercises. Back stretch in sitting position (2 min)	Video: Strengthening of upper limbs (4 min). Balance and knee flexion exercises (2 min)	Videos: 'Yoga' sun salutation. Three times upon awakening (1.3 min)
Psychoeducation	Audio: Mindfulness. Breathing (6 min)	Audio: Mindfulness. Breathing (6 min)	Audio: Mindfulness\ACT. Internal events: Thoughts. Leaf dynamics.	Audio: Mindfulness. Body scanner.	Audio: Mindfulness\ACT. Internal events: Emotions.	Audio: Mindfulness. Adaptation of the spot on the face metaphor
	Audio: ACT. Adaptation of the garden metaphor (4 min)	Audio: Mindfulness. Body scanner (6 min)	Audio: ACT. Bus metaphor	Audio: Grey clouds exercise	Audio: Mindfulness\ACT. Internal events: Thoughts. Leaf dynamics	Audio: Mindfulness\ACT. Internal events: Emotions.

	Questionnaire: Written exercise to gain "insight" on metaphor.<br>What plants are in your first garden?<br>What are the biggest ones?<br>Have you tried to pull out the weeds? How? What are the short-term consequences? What happens to the plants in your garden if you just pluck the weeds?<br>Is there an alternative to pulling the weeds? How is your garden today? Can you take care of the garden even if it has weeds? Do you want to improve your garden? What could you do ? What plants could you water? How would you water them?	Questionnaire Part 1: Value the importance you give to your vital areas.<br>On a star chart (scale 1-7). Family, Couple; Competence: work, learning, volunteering;<br>Health: exercise, food, sleep; Leisure; Friendship: social life.<br>Questionnaire Part 2: Value your current garden. How is your life at the moment?<br>On a star-like chart (scale 1-7). Family, Couple; Competence: work, learning, volunteering;<br>Health: exercise, food, sleep; Leisure; Friendship: social life.<br>Visual feedback from the two stars: the life I want and the one I have.	Questionnaire. Which passengers make you feel uncomfortable?<br>Have you tried to get them off the bus?<br>What happens when you tell them to get off?<br>Is there a life without uncomfortable passengers?<br>Where is your life bus going right now?<br>Who do you want to direct your life? The passengers (pain) or you?<br>Can you choose the direction of the bus even with passengers? Is it impossible or difficult?<br>Where do we want to go with the bus?<br>Let's think about the intermediate stops that will take us to that place.<br>These stops are the goals.	Questionnaire Part 1: Select goals by vital areas.<br>Family, Couple; Competence: work, learning,<br>volunteering; Health: exercise, food, sleep, etc.;<br>Leisure; Friendship: social life.<br>Questionnaire Part 2: Written exercise:<br>Select concrete actions to achieve the proposed goals.	Part 1: Select 3 activities to do during the week according to the proposals of the previous week.<br>Start with the easiest ones.<br>Part 2: Perform 3 of the selected actions throughout the week.	Questionnaire: Select 7 valuable activities that you can do during the week.<br>Perform 1 of the selected actions each day.
Pharmacological	Relate each medication to its type: analgesic (paracetamol, morphine, codeine);<br>anti-inflammatory (naproxen, ibuprofen, celecoxib)	Relate each medication to its type: analgesic (paracetamol, morphine, tramadol);<br>anti-inflammatory (naproxen, ibuprofen, diclofenac)	Relate each medication to its type: analgesic (metamizol, tramadol, dexketoprofen, codeine); anti-inflammatory (naproxen, diclofenac, colecoxib)	Relate each medication to its type: analgesic (metamizol, tramadol, dexketoprofen, codeine); anti-inflammatory (naproxen, diclofenac, colecoxib)	Relate each medication to its type:<br>analgesic (metamizol, tramadol, dexketoprofen, codeine); anti-inflammatory (naproxen, diclofenac, colecoxib)	Relate each medication to its type:<br>analgesic (metamizol, tramadol, dexketoprofen, codeine); anti-inflammatory (naproxen, diclofenac, colecoxib)
	Video: Explanation of pain scale, types and treatment of nociceptive,<br>neuropathic and mixed pain (2 min)	Video: Explanation of pain scale, types and treatment of nociceptive, neuropathic and mixed pain (2 min)	Video: Explanation of pain scale, types and treatment of nociceptive, neuropathic and mixed pain (2 min)	Video: Explanation of pain scale, types and treatment of nociceptive, neuropathic and mixed pain (2 min)	Video: Explanation of pain scale,<br>types and treatment of nociceptive,<br>neuropathic and mixed pain (2 min)	Video: Explanation of pain scale, types and treatment of nociceptive,<br>neuropathic and mixed pain (2 min)
	Questionnaire: Select side effects<br>Paracetamol (hypertension), anti-inflammatory (hemorrhages) and tramadol (hypertension)	Questionnaire: Select side effects<br>Paracetamol (hypertension), anti-inflammatory (hemorrhages) and tramadol (hypertension)	Questionnaire: Select side effects<br>Paracetamol (hypertension), anti-inflammatory (hemorrhages) and tramadol (hypertension)	Questionnaire: Select side effects<br>Paracetamol (hypertension), anti-inflammatory (hemorrhages) and tramadol (hypertension)	Questionnaire: Select side effects<br>Paracetamol (hypertension), anti-inflammatory (hemorrhages) and tramadol (hypertension)	Questionnaire: Select side effects<br>Paracetamol, (hypertension), anti-inflammatory (hemorrhages) and tramadol (hypertension)

Health Assets	Audio: Explanation of individual's own assets (1 min)	Audio: Associational assets (1 min)	Audio: Organizational and institutional assets (1 min)	Audio: Physical assets (1 min)	Audio: Cultural assets (1 min)	Audio: Economic assets (1 min)
	Video: Explanation of own assets (social competence, strengths, emotions, reasoning, skills) (1 min)	Video: Explanation of the health assets of associations, discover those assets (sports clubs, volunteer groups, etc.) (1min)	Video: Explanation of the health assets of institutions, discover those assets (healthcare centers, educational centers, local government services) (1 min)	Video: Explanation of physical assets, discover those assets (green spaces, sports facilities) (1 min)	Video: Explanation of cultural assets, discover those assets (theatre, exhibitions, cinema) (1min)	Video: Explanation of economic assets, discover those assets (local commerce, occupational activities) (1min)
	Reflection: What makes me healthier? What do I have or is there around me that can help me? Identify own assets.	Reflection: What makes me healthier?<br>What do I have or is there around me that can help me? Identify the geolocation of associations' economic and cultural assets.	Reflection: What makes me healthier? What do I have or is there around me that can help me? Identify the geolocation of organizations and institutions' assets and resources that help maintain health.	Reflection: What makes me healthier? What do I have or is there around me that can help me? Identify the geolocation of physical assets and resources that help maintain health.	Reflection: What makes me healthier? What do I have or is there around me that can help me? Identify the geolocation of cultural and leisure assets and resources that help maintain health.	Reflection: What makes me healthier?<br>What do I have or is there around me that can help me? identify the geolocation of individual, physical, institutional, associative, economic and cultural assets.
